# Sensitive and semiquantitative detection of soil-transmitted helminth infection in stool using a recombinase polymerase amplification-based assay

**DOI:** 10.1371/journal.pntd.0009782

**Published:** 2021-09-13

**Authors:** Jason L. Cantera, Heather N. White, Matthew S. Forrest, Oliver W. Stringer, Vicente Y. Belizario, Helen L. Storey, Eugenio L. de Hostos, Tala de los Santos

**Affiliations:** 1 PATH, Seattle, Washington, United States of America; 2 TwistDx Limited, Norman Way, Cambridge, United Kingdom; 3 Department of Parasitology, College of Public Health, University of the Philippines, Ermita, Manila, Philippines; Christian Medical College, Vellore, INDIA

## Abstract

**Background:**

Soil-transmitted helminths (STHs) are parasitic nematodes that inhabit the human intestine. They affect more than 1.5 billion people worldwide, causing physical and cognitive impairment in children. The global strategy to control STH infection includes periodic mass drug administration (MDA) based on the results of diagnostic testing among populations at risk, but the current microscopy method for detecting infection has diminished sensitivity as the intensity of infection decreases. Thus, improved diagnostic tools are needed to support decision-making for STH control programs.

**Methodology:**

We developed a nucleic acid amplification test based on recombinase polymerase amplification (RPA) technology to detect STH in stool. We designed primers and probes for each of the four STH species, optimized the assay, and then verified its performance using clinical stool samples.

**Principal findings:**

Each RPA assay was as sensitive as a real-time polymerase chain reaction (PCR) assay in detecting copies of cloned target DNA sequences. The RPA assay amplified the target in DNA extracted from human stool samples that were positive for STH based on the Kato-Katz method, with no cross-reactivity of the non-target genomic DNA. When tested with clinical stool samples from patients with infections of light, moderate, and heavy intensity, the RPA assays demonstrated performance comparable to that of real-time PCR, with better results than Kato-Katz. This new rapid, sensitive and field-deployable method for detecting STH infections can help STH control programs achieve their goals.

**Conclusions:**

Semi-quantitation of target by RPA assay is possible and is comparable to real-time PCR. With proper instrumentation, RPA assays can provide robust, semi-quantification of STH DNA targets as an alternative field-deployable indicator to counts of helminth eggs for assessing infection intensity.

## Author summary

More than 1.5 billion people are infected with parasitic intestinal worms called soil-transmitted helminths. Infection is transmitted by helminth eggs in human feces, which contaminate soil in areas with poor sanitation. Adverse health effects include physical and cognitive impairment in children. A key strategy to control infection is periodic mass drug administration for populations with a high prevalence of disease based on the results of diagnostic testing. The current microscopy method for detecting infection, however, has limited ability to detect disease as the intensity of infection decreases with repeated mass drug administration. To address limitations of current diagnostic methods, we developed a novel technique to diagnose infections, including those at very low levels of intensity, by detecting helminth DNA in stool samples. Our initial studies suggest that the new diagnostic technique reliably detects the presence of intestinal worms, even at low intensities of infection, and may be more useful than currently available diagnostic tools for guiding the use of periodic mass drug administration to eliminate disease in low-resource settings.

## Introduction

Soil-transmitted helminths (STHs)—including *Ascaris lumbricoides*, *Trichuris trichiura*, and two hookworm species, *Ancylostoma duodenale* and *Necator americanus*—remain a massive global health problem, affecting more than 1.5 billion people each year, particularly among poor populations [1]. STH infections are transmitted by helminth eggs present in human feces, which contaminate soil in areas with poor sanitation. STH infections have significant harmful effects on the health and well-being of individuals, especially children, and reduce economic productivity in many countries [2,3]. For children, STH infections may lead to malnutrition, anemia, abdominal pains, stunted growth, and poor cognitive development.

The main element of the global STH control strategy is reducing morbidity in the most at-risk populations—such as preschool-age children, school-age children, and women of reproductive age—through mass drug administration (MDA) of deworming drugs, such as mebendazole and albendazole [4]. Program decisions for STH control rely on detecting the presence and quantifying the load of helminth eggs in stool. The prevalence of infection informs the frequency of MDA for a given population and measuring the intensity of infection helps to assess the efficacy of MDA and other interventions for reducing morbidity [5,6]. Thus, reliable prevalence data through surveillance is critical to justify continued or reduced frequency of or discontinue MDA, ensuring that MDA is not stopped prematurely before morbidity control targets have been reached, and reduce the possibility of worms developing resistance to drugs used for MDA.

Successful MDA programs lead to low-intensity infections, and this increases the need for accurate STH diagnosis to evaluate the impact of MDA and sustain the overall benefits. Inaccurate diagnosis may underestimate the prevalence and intensity of infection and prevent a reliable estimation of MDA impact [7]. Monitoring the effectiveness of these programs and other interventions relies heavily on the accuracy of diagnostic tests to determine the presence and level of infection [8].

Although far from perfect, the current gold standard test for diagnosing STH infection relies on microscopic examination of stools for STH eggs by the Kato-Katz (KK) method [9] or by World Health Organization-recommended mini-FLOTAC [10] methods. Although both copromicroscopy methods are adequate to support early-stage program decisions when the prevalence and intensity of infections are moderate to high, they lack sensitivity with light-intensity infections, especially after MDA has reduced infections to low levels [5,11–13]. Also, while the presence of eggs in stool is a good indicator of morbidity at high worm burdens, it is not the best indicator for future transmission risk with lower worm burdens [14]. The availability of diagnostic tools that are more sensitive than current methods for detecting low-intensity infections will improve the ability of control programs to determine whether program goals have been achieved and whether and when to reduce or stop MDA.

Nucleic acid amplification tests (NAATs) such as polymerase chain reaction (PCR)-based technologies are attractive alternatives to existing microscopy and serological methods; they are highly sensitive and specific and allow the detection and discrimination of STHs and a wide variety of pathogens [15–20]. Although PCR-based technologies are reliable, they have not been widely used in low-resource settings [21] because of the requirements for complex, expensive equipment and highly skilled personnel. Isothermal NAATs offer the potential to eliminate the need for expensive thermal cyclers, and several isothermal NAAT methods have been described for STH diagnosis [22–25]. The problem with these assays is the difficulty to detect multiple targets without also increasing the probability of nonspecific amplifications due to the number of oligonucleotide primers needed per target. Thus, new rapid, sensitive, and cost-effective methods for detecting STH infections using other molecular diagnostic tools that can be adapted to low resource, low infrastructure, non-laboratory field conditions are desirable [9] to help STH control programs achieve their goals.

Recombinase polymerase amplification (RPA) is a rapid, highly sensitive, and specific isothermal NAAT that uses several enzymes to amplify DNA or RNA. These enzymes include recombinases that form complexes with oligonucleotide primers and pair the primers with homologous sequences in the DNA; a single-stranded DNA binding protein that binds to the displaced DNA strand and stabilizes the resulting D-loop; and a strand-displacing DNA polymerase that initiates DNA amplification when the primer binds to the target DNA sequence [26]. RPA offers many features that make it more attractive than other PCR-based tests for use in low-resource settings. For example, RPA occurs rapidly, typically in less than 15 minutes, even with very few copies of DNA [26], so results are available much more quickly than with PCR. Also, RPA operates at a lower temperature range of 35–40°C, negating the need for a complex and relatively expensive thermal cycler [27–29]. It has also demonstrated an ability to quantify targets comparable to real-time PCR [30–32]. RPA assays have already been developed for a few parasitic protozoa and other neglected tropical diseases, including giardiasis [33], toxoplasmosis [34], schistosomiasis [35,36], fasciola [37], chikungunya [38], and leishmaniasis [39,40].

We developed a sensitive, field-deployable, RPA-based molecular tool (herein named Dx4STH) [8] for diagnosis of STH infection to improve decision-making on the frequency and duration of MDA to reduce the transmission and associated morbidity of STH infections. The RPA tests target four species of helminths using their ribosomal RNA gene targets. We assessed the performance of the RPA tests using clinical stool samples and compared the results to those of a STH real-time PCR assay and to copromicroscopy analyses.

## Methods

### Ethics statement

Collection of the stool samples were conducted during the SCH-ELISA 2 fieldwork in June-July 2015 in Compostela Valley, Philippines under ethics review clearance code UPMREB-2013-NIH-P2-O47. Written informed consent was obtained from the parent prior to collection.

### Plasmid DNA standards, genomic DNAs, and *Ascaris suum* eggs

Target gene sequences specific to *A*. *lumbricoides*, *A*. *duodenale*, and *N*. *americanus* (S1 Table) were PCR-amplified from DNA extracted from STH-positive stools, while a DNA fragment from the *T*. *trichiura* genome (nucleotide position 71488–71604, GenBank HG805809.1) was synthesized and obtained from Integrated DNA Technologies (Coralville, IA). The PCR amplicons and the synthetic gene were cloned into pCR4-TOPO cloning vector (Invitrogen, Carlsbad, CA), and the inserts were verified by Sanger sequencing. The concentrations of purified plasmid DNA (pAl, pTt, pAd and pNa) were measured using Nanodrop ND-1000 (NanoDrop Instruments, Wilmington, DE). Appropriate dilutions of plasmid stocks were made in TE buffer to obtain working concentrations from 10^0^ to 10^8^ copies/μL. Background DNA was added to RPA and real-time PCR reactions at 150 ng/μL final concentration. These plasmid DNA standards were used for assay development and performance testing.

DNA from non-target enteric pathogens (NTEPs)—including entero-aggregative and enteropathogenic *E*. *coli*, *Salmonella typhimurium*, *Shigella flexneri*, *Listeria monocytogenes*, *Campylobacter jejuni*, *Clostridium difficile*, *Cryptosporidium parvum*, and *Giardia*—were obtained from PATH’s DNA collection. *Entamoeba histolytica* was procured from ZeptoMetrix Corp. (Buffalo, NY), and *Schistosoma japonicum* DNA was extracted from a stool sample. *Ascaris suum* eggs were obtained from Excelsior Sentinel, Inc. (Trumansburg, NY), and the count was determined using a hemocytometer.

### Stool samples, DNA extraction, and real-time PCR assays

Deidentified stool specimens from school-age children in STH-endemic regions in the Philippines were obtained from Dr. Vicente Belizario of the University of the Philippines Manila. Stool samples for a spiking experiment were procured from BioIVT (Westbury, NY). Total genomic DNA was extracted from 200 mg of stool using the QIAamp PowerFecal DNA kit (QIAGEN, Valencia, CA) following the manufacturer’s instructions, and eluted in 200 μL elution buffer.

Real-time PCR primers and probes for each STH species, including the amplification conditions are listed in S1 Table.

### RPA assay development and optimization

#### RPA primers and probe selection

The target genes for PCR detection of STHs [15,17,41] were used for RPA assay design. For each target species, at least two probes and about 20 to 30 primer pairs per probe were designed according to guidelines (www.twistdx.co.uk). For optimal RPA performance, candidate primers and probe pairings were screened for each STH species in duplicate reactions using either 50 or 20 plasmid DNA copies per reaction using TwistAmp exo kits (TwistDx, Cambridge, UK) following the manufacturer’s recommended protocols. We selected the primers and probe sets that produced the shortest amplification times, steep fluorescence increases when template was present, a flat fluorescence baseline in the negative control, and consistent fluorescent curves between replicate reactions. Table 1 lists the optimal primers and probe for each target.

**Table 1:** RPA primers and probes designed in this study.

Assay name	Target organism	Oligo name	Sequence (5’ —> 3’)	Nucleotide position	Amplicon size (bp)	Target gene	GenBank acc. No.
Al-RPA	*Ascaris lumbricoides*	Al-F5	ACACAAATGTGGTGATGTAATAGCAGTCGGCGGTT	81–115	124	ITS1	AB571301.1
		Al-R7	AGCGGCATGCCTTTCTAACAAGCCCAACATGCCAC	205–171			
		Al-P3	TTTTTTTGGCGGACAATTGCATGCGATTFGHQATGTGTTGAGGGA	120–164			
Ad-RPA	*Ancylostoma duodenale*	Ad-F3a	GCACTTGCTTTTAGCGATTCCCGTTCTAGATCA	562–594	126	ITS2	EU344797.1
		Ad-R1c	AAGTCGGTAAACGATTCAGCAGCAACAACGAGTTT	688–654			
		Ad-P1-2	CCGTTCTAGATCAGAATATATTGCAACATGFAHGQTAGCTGGCTAGTTTGC	582–632			
Na-RPA	*Necator americanus*	Na-F4	GTAGCTTGTGGACAGTACTCTCACCGAGTA	18–47	116	ITS2	AJ001599.1
		Na-R3-6	TGTGTTCTTCACTTAAACGGGAATTGCTGAACAC	134–101			
		Na-P3-1	TTGTTGAACACTGTTTGTCGAACGGTACFTHCQCTGTACTACGCATTG	48–85			
Tt-RPA	*Trichuris trichiura*	Ttrpt-F5	GTTTCATAGTTGATCTTCAGATTCACGGGTTTGGC	71489–71523	117	unk	HG805809.1
		Ttrpt-R2	TAACAATTTGCTCATCCATCCGTTGGTAGGGCATT	71556–71588			
		Ttrpt-PR1	CCATCCGTTGGTAGGGCATTTTGAAGTTFHCQTACAGATACATCC	71529–71573			

*Note*: TwistAmp exo Probe contains an abasic nucleotide analogue (H), fluorophore (F) and a corresponding quencher (Q). F and Q replaced T residue found within the corresponding target sequence. All probes are blocked from any potential polymerase extension by a 3’–modification group (C3-spacer). The analogue, H, is the cleavage site of Exonuclease III present in the TwistAmp exo kit.

Each singleplex RPA reaction contained 0.48 μM each of forward and reverse primers, 0.12 μM probe, 150 ng of background stool DNA, 2 μL of template DNA, and 1X of supplied rehydration buffer. Negative control (NC) reactions were included using background stool DNA instead of the target DNA. A total of 47.5 μL of master mix was aliquoted into each reaction tube containing the freeze-dried pellet, and then 2.5 μL of 280 mM magnesium acetate (MgAc) was added to the lid of the tube. Tubes were spun down to mix the MgAc into the solution to initiate the reaction. RPA reactions were monitored in real-time via fluorescent detection for FAM and ROX using either Twista (TwistDx, Cambridge, UK) or an ISO-T8 device (Axxin, Victoria, Australia) for 15 min at 40°C with a 30 sec measurement interval and a manual mixing step at 4 min incubation.

#### Duplex/triplex assay development

**Fig 1:**
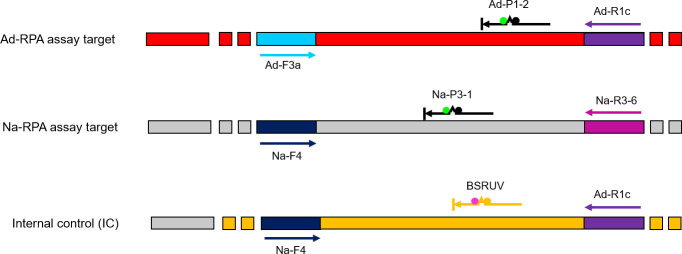
Development of an internal control (IC). A short section of *Bacillus subtilis* gDNA was amplified using primers tailed with Ad- or Na-RPA primer sequences, and the resulting PCR product was cloned into a plasmid. The plasmid construct (IC) contains the Na-F4 (dark blue) and Ad-R1c (purple) primer binding sites. BSRUVB probe designed to target the *B*. *subtilis* DNA fragment [42] is shown in yellow, while Na and Ad probes are in black. In RPA assay, the IC is amplified by the Na-F4 and Ad-R1c primers and the amplicon detected by BSRUV.

Duplex/triplex RPA assays were customized at TwistDx (Cambridge, UK) using the optimized primers and probe set for each target species. The assays were further optimized by testing different primer biases to improve performance. Different RPA protein ratios were compared to see whether a custom formulation would improve the limit of detection and speed of the reactions. Finally, different ratios of the individual assays were combined to maximize their performance. For internal control (IC), a novel plasmid DNA containing a *Bacillus subtilis* DNA fragment was developed (Fig 1), and then lyophilized into the Ad-RPA/ Na-RPA reactions along with its corresponding probe that specifically target the cloned *B*. *subtilis* DNA.

The customized, two-tube duplex/triplex RPA assay, referred to as Dx4STH-1 (duplex, for detecting *A*. *lumbricoides* and *T*. *trichiura*) and Dx4STH-2 (triplex, for *A*. *duodenale–N*. *americanus–*IC), contains a lyophilized pellet with all the proteins, dNTPs and oligonucleotides needed to run an RPA reaction. For testing, 2 μL of template DNA was added into 23 μL water and 25 μL supplied rehydration buffer with MgAc, and then aliquoted into each reaction tube containing the customized RPA pellet. RPA reactions were monitored in real-time using fluorescent detection as described above.

#### Analytical performance testing

RPA assays were tested using DNA extracts from STH-positive stools and using genomic DNA from NTEPs at 1 ng per reaction to test their specificity. The analytical sensitivity/ limit of detection of each assay was assessed using four plasmid DNA (pAl, pTt, pAd and pNa), each harboring the target DNA region at levels ranging from 5 to 1000 target copies per reaction in 10 or 20 replicate reactions per level. The limit of detection was determined using probit analysis.

#### Semi-quantification algorithm

Raw fluorescence data from RPA assays were exported into Excel for analysis. Threshold time (T_t_), which represents the time in minutes at which the fluorescence signal crosses the fluorescence threshold (F_t_) and is therefore analogous to threshold cycle in real-time PCR, was manually determined from each positive amplification. F_t_ for each probe was defined as the maximum fluorescence signal (from time 0 to 15 minutes) in a NC reaction + zSD, where NC is the negative control, z is an arbitrary value set between 10 and 100, and SD is the standard deviation of the mean fluorescence signal for each probe. A sample was positive if the fluorescence generated within a reaction was significantly above the background fluorescence and crossed F_t_.

To correlate amplification time with target concentration, RPA was run using plasmid DNA standards, after which T_t_ was determined and then plotted against plasmid concentration to construct a standard curve. The procedure was conducted four times to establish reproducibility of results, creating 12 sample data points per concentration.

### Egg-spiking and detection of light and moderate intensities of infection

To demonstrate feasibility of estimating infection intensities, 200-mg aliquots of STH-negative stools were spiked with *A*. *suum* eggs at various levels (i.e., 15,000, 5,000, 1,500, 500, 150, and 50 eggs per gram [EPG]) in five replicates per level corresponding to medium and light intensities of infection. *A*. *suum* was used as model because of the availability of quantified eggs from a commercial source, its low genetic divergence with *A*. *lumbricoides* [43], and its DNA can be detected by both Al-RPA and real-time PCR assays. The total DNA from each spiked stool was extracted using a column-based DNA extraction that involved chemical and mechanical bead-beating steps that may be appropriate even for the tough eggs of *T*. *trichiura* [19]. The extract was subjected to Al-RPA and real-time PCR. Al-RPA was used instead of Dx4STH-1 as the two assays have similar performance in detecting *A*. *lumbricoides*. The T_t_ value from each *Ascaris*-positive sample was estimated from the amplification curve and then plotted against the concentration of egg in stool.

### Assay performance using stool samples

One hundred stool specimens were used to assess the performance of the RPA assays. Total DNA was extracted from 200 mg of all 100 samples by the method described above. A 2-μL aliquot of each extract was analyzed using Dx4STH-1, Dx4STH-2, and real-time PCR assays. The sample was considered positive if either KK or PCR were positive. Samples were scored RPA-positive when the fluorescence signal crossed T_t_. Positive, negative, and overall agreements were calculated to assess concordance between the RPA, real-time PCR, and Kato-Katz method using equations as previously described [44]. IC in all stool DNA extracts were amplified, suggesting minimal effect of fecal confounders.

Analysis of data was performed using Minitab 17.1.0 and Excel 2016. Correlations were estimated from Pearson correlation coefficients in R. Kappa statistics, which account for the possibility that concordance may occur by chance, were used for comparison of each assay for each species.

## Results

### Development and optimization of RPA assays for detecting STH

**Fig 2:**
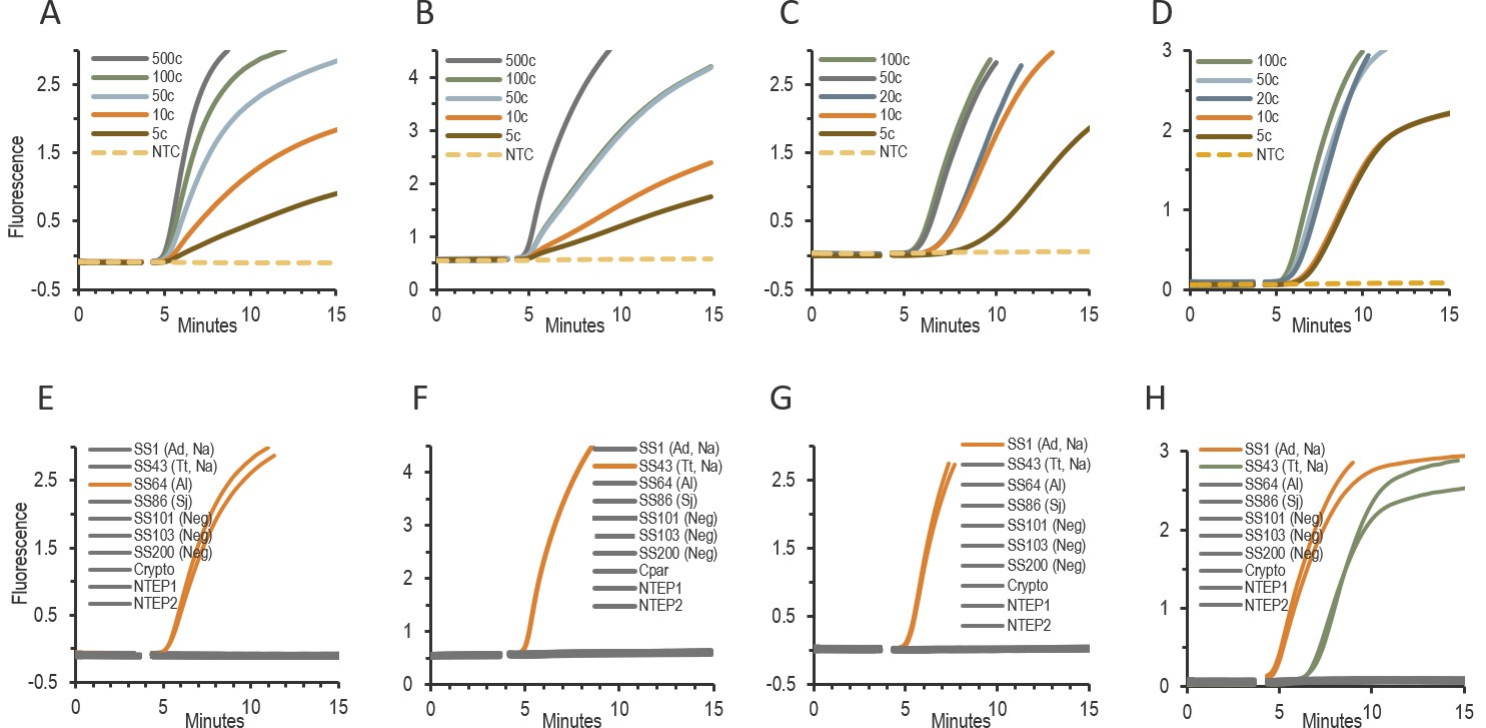
**Amplification curves from Al-RPA (A and E), Tt-RPA (B and F), Ad-RPA (C and G) and Na-RPA (D and H).** Raw fluorescence intensity of labeled probes used for the detection of plasmids pAl, pTt, pAd and pNa at varying copies per reaction (A, B, C and D), or total DNA extracted from STH-positive stool samples (SS) and genomic DNAs from non-target enteric pathogens (E, F, G and H). Al, *A*. *lumbricoides*; Tt, *T*. *trichiura*; Ad, *A*. *duodenale*; Na, *N*. *americanus*; Sj, *Schistosoma japonicum*; Crypto, *Cryptosporidium parvum*; Neg, STH-negative; NTC, no template control. NTEP, non-target enteric pathogen; NTEP1 included DNA from *Clostridium difficile* 630, *Campylobacter coli*, *Escherichia coli* O104, *Listeria monocytogenes*, *Salmonella* sp. MDR, *Shigella flexnerii*, and *Entamoeba histolytica*; NTEP2 included *Salmonella typhimurium*, *E*. *coli* O145, and *C*. *difficile* 027.

Four singleplex RPA assays were developed and optimized through extensive primer and probe screening. With use of the optimal RPA primers and probe set for each STH species, all four assays detected less than 10 target copies per reaction within 8 min of incubation at 40°C. When assessed for specificity using DNA extracts from STH-positive stools and genomic DNAs from unrelated pathogens commonly found in the gastrointestinal tract or in clinical stool specimens, each RPA assay amplified only the intended target DNA. Fig 2 shows the amplification curves from the four RPA assays using either plasmid DNA at varying target copies (2A, 2B, 2C, and 2D) or DNA extracts (2E, 2F, 2G, and 2H).

To minimize the number of RPA reactions needed for testing each sample, the assays were pooled into two multiplexed RPA assays: Al-RPA was combined with Tt-RPA (Dx4STH-1), and Ad-RPA with Na-RPA and IC (Dx4STH-2). The conditions were further optimized by varying the ratio of the various components in each assay to ensure that the performance characteristics of the multiplex RPA assays were equivalent to those of singleplex RPA assays.

### Analytical sensitivity and specificity

To verify if performance characteristics of the multiplex assays is equivalent to the singleplex, the analytical specificity and sensitivity of Dx4STH-1 and Dx4STH-2 were compared to those of the individual RPA assays. Similar to that of singleplex assays, Dx4STH-1 and Dx4STH-2 detected only their intended targets. Dx4STH-1 has a limit of detection (LOD) of 45.9 and 16.3 target copies for *A*. *lumbricoides* and *T*. *trichiura*, respectively, whereas Dx4STH-2 has an LOD of 10.0 and 14.0 target copies for *A*. *duodenale* and *N*. *americanus* (Table 2). These values were not significantly different from those for the individual assays (t-test *P* = 0.097). The NC did not show amplification, but the IC in Dx4STH-2 was amplified in all cases with a mean T_t_ of 6.1 ± 0.6 min. We observed a minimal reduction in fluorescent signals in IC at higher target concentration (10^5^ and 10^6^ target copies), but this observation is not surprising because of competition for primers during amplification [31]. Regardless, this did not affect target amplification in all concentrations tested.

**Table 2:** Limit of detection of each RPA assay as determined by probit analysis using 10 to 20 replicates per concentration tested.

Method	Limit of detection (95% CI)			
	*A*. *lumbricoides*	*T*. *trichiura*	*A*. *duodenale*	*N*. *americanus*
RPA (Dx4STH-1, Dx4STH-2)	45.9	16.3	< 10.0	14.0
RPA (singleplex)	11.9	6.7	7.3	10.9
PCR	62.6	36.0	65.6	34.6

*Note*: Data sets of each assay run were determined from plasmid standard panel with defined amount ranging from 10 to 10^5^ copies in 150 ng of background stool DNA at 10 (singleplex) to 20 replicates (Dx4STH) per amount. Probit regression analysis was conducted using Minitab 17.1.0. The LOD of Dx4STH-1 and Dx4STH-2 was not significantly different from that of the corresponding singpleplex assays (t-test *P* = 0.097). Real-time PCR assays on the same plasmid DNA standard without background DNA were not as sensitive as RPA assays in either multiplex (t-test *P* = 0.045) or singleplex format (t-test *P =* 0.034).

In comparison, real-time PCR assays on the same plasmid DNA standard containing background DNA showed inconsistent results, with observed higher C_q_ values (>3 C_t_) and an LOD at 10^4^ copies. When the background DNA was removed in the reaction, the LOD of each real-time PCR assay was higher than that of the corresponding RPA assay in either multiplex (t-test *P* = 0.045) or singleplex format (t-test *P =* 0.034), suggesting that RPA assays detected their respective targets at lower levels than real-time PCR, and were more robust in the presence of background DNA found in stool samples. It is also possible that the plasmids used as standard for real-time PCR are in supercoil form, which may have suppressed PCR by decreasing the efficiency for primer binding and elongation in a PCR reaction [45,46], resulting to higher LOD values for real-time PCR than RPA.

### Linearity, accuracy, precision, and detection of low levels of eggs

**Fig 3:**
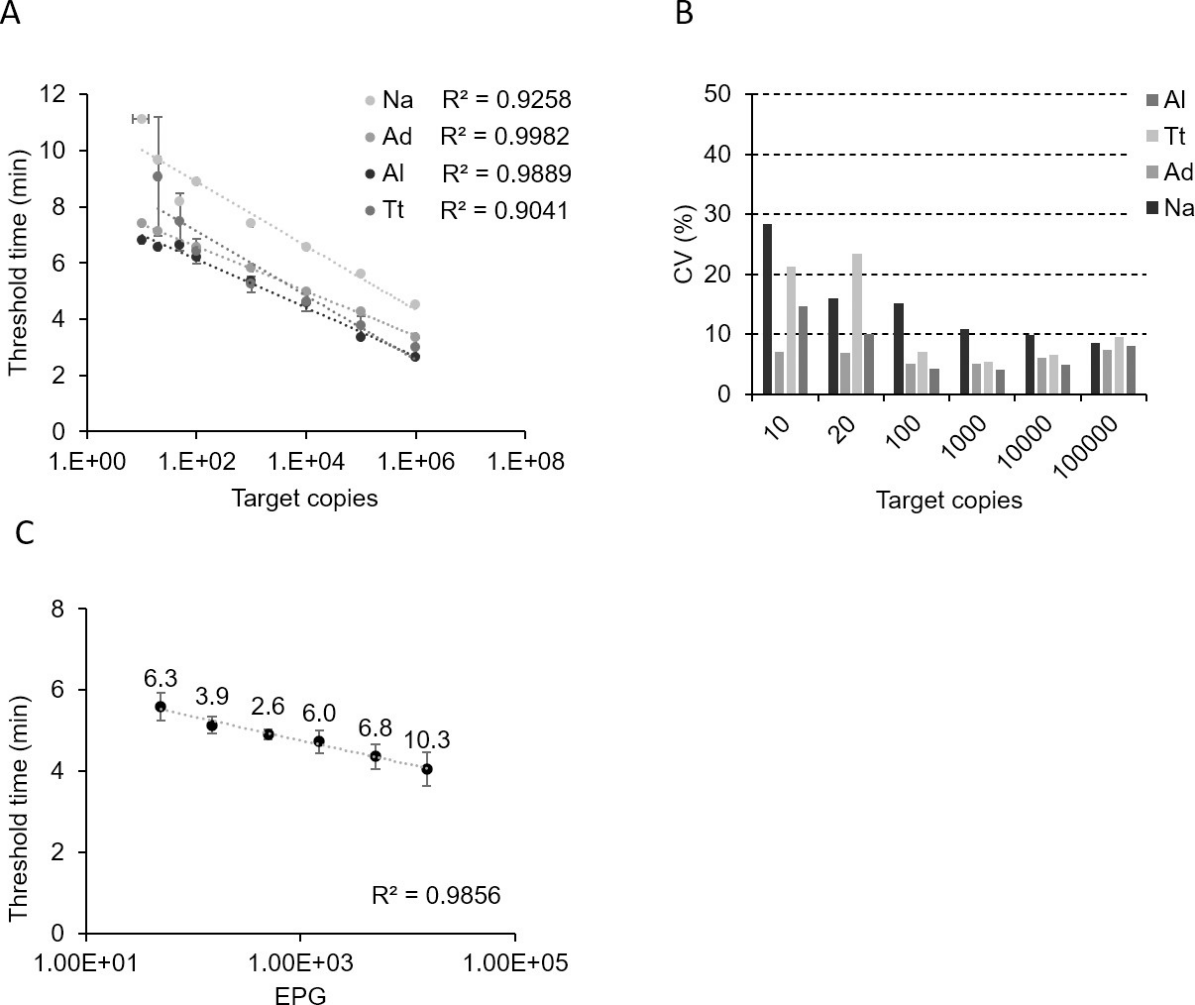
(A) Standard curves generated from plasmids with defined concentrations of 1000, 500, 100, 50, 20, and 10 copies per reaction using Dx4STH-1 and Dx4STH-2. (B) Coefficients of variation of T_t_ values from four independent runs were calculated to determine accuracy and assay repeatability. Dx4STH-1 and Dx4STH-2 have a wide operating range for quantifying target DNA, spanning 5 to 7 logs, with corresponding amplification time of 2–4 min at 10^6^ copies and 7–11 min at 10 copies per reaction. (C) Al-RPA standard curve using T_t_ values from each positive amplification and plotted against target copies present in DNA extracted from stool samples that were spiked with *Ascaris suum* eggs at varying levels (5 replicates/level) corresponding to moderate and light intensity infections (Pearson’s *r =* 0.9856). Note: Al, *A*. *lumbricoides;* Tt, *T*. *trichiura;* Ad, *A*. *duodenale;* and Na, *N*. *americanus*. EPG, eggs per gram.

To determine if T_t_ correlates with target copies present in the reaction, we analyzed the T_t_ in each RPA experiment using the quantification algorithm as described. The T_t_ for Dx4STH-1 and Dx4STH-2 experiments increased with decreasing DNA concentration. Standard curves generated using the plasmid standards showed high R^2^ values ranging from 0.904 to 0.998 (Fig 3A), suggesting excellent correlations between T_t_ and input DNA. We also assessed the accuracy and precision (repeatability) of the assay by calculating the coefficient of variation (CV) of T_t_ values in four independent runs. The CVs were less than 10% for 10^6^ down to 10 copies for *A*. *duodenale*, and 10^2^ copies for *A*. *lumbricoides* and *T*. *trichiura* (Fig 3B). Higher CVs (>10%) at lower target concentrations were observed for *N*. *americanus*, which was not surprising because this assay was the most affected by the IC.

Using a cutoff value of 20% CV, we demonstrated that Dx4STH-1 and Dx4STH-2 have a wide operating range for quantifying target DNA, spanning at least 5 logs, with a corresponding amplification time of 2–4 min at 10^6^ copies and 7–11 min at 10 copies per reaction. Additional testing is needed to verify the performance of these assays.

To demonstrate if RPA can detect low-intensity infection, we analyzed the DNA extracted from stools spiked with *A*. *suum* eggs at levels corresponding to moderate and light intensity infections. Results showed positive amplification from 15,000 EPG (9/9 positives) down to 50 EPG (9/9 positives) using Al-RPA. We also observed amplification in stool extracts with 25 EPG of *A*. *suum* eggs in a separate experiment, highlighting the probability that Al-RPA can detect an infected individual with light-intensity infection, and the probability decreased at <50 EPG. The mean T_t_ value correlated well with EPG (R^2^ = 0.9856) (Fig 3C).

### Diagnostic accuracy of Dx4STH-1 and Dx4STH-2 and estimation of intensity of infection in stool

A total of 100 de-identified stool specimens with single or mixed STH infections based on the KK method [*A*. *lumbricoides* (n = 22), *T*. *trichiura* (n = 34), hookworms (n = 17), and uninfected (n = 36)] were used to assess the diagnostic performance of the assays. Real-time PCR assays showed that 19, 29, and 22 samples were positive for *A*. *lumbricoides*, *T*. *trichiura*, and hookworms, respectively. When these samples were tested using Dx4STH-1 and Dx4STH-2, 19, 28, and 20 were positive for *A*. *lumbricoides*, *T*. *trichiura*, and hookworms. In all stool samples tested, amplification of the IC by RPA and real-time PCR was detected, suggesting that there was no evidence of inhibition of amplification in any of the DNA extracts. Using the results of real-time PCR as the comparator in determining infection status, we found that Dx4STH-1 showed 100% sensitivity and 100% specificity for *A*. *lumbricoides* and 97% sensitivity and 100% specificity for *T*. *trichiura*. Dx4STH-2, by comparison, exhibited 91% sensitivity and 97% specificity for the hookworm species.

We used Kappa agreement statistics to compare the results with various diagnostic techniques. Overall, the results of Dx4STH-1 and Dx4STH-2 were in very good agreement with those of real-time PCR assay for detecting *A*. *lumbricoides* (κ = 1.00), *T*. *trichiura* (κ = 0.98), and the two hookworm species (κ = 0.88). Use of the KK method as comparator, by contrast, led to 7 false positives and 11 false negatives. Table 3 shows the diagnostic accuracy of the assays.

**Table 3:** Sensitivity and specificity of RPA against real-time PCR or Kato-Katz as comparator method.

STH	Test results	PCR +	PCR -	Kappa*	Sensitivity	Specificity
RPA vs PCR						
*A*. *lumbricoides*	RPA +	19	0	1.00	100%	100%
	RPA -	0	81			
*T*. *trichiura*	RPA +	28	0	0.98	96.6%	100%
	RPA -	1	71			
*A*. *duodenale/*	RPA +	20	2	0.88	90.9%	97.4%
*N*. *americanus*	RPA -	2	76			
RPA vs KK						
*A*. *lumbricoides*	RPA +	17	5	0.79	89.5%	93.8%
	RPA -	2	76			
*T*. *trichiura*	RPA +	27	5	0.86	96.6%	91.5%
	RPA -	1	67			
*A*. *duodenale/*	RPA +	18	1	0.85	68.2%	97.4%
*N*. *americanus*	KK -	4	77			

* The agreement between the diagnostic methods was assessed using κ-statistics. The κ-statistics were interpreted as < 0.20, poor agreement; 0.21–0.40, fair agreement; 0.41–0.60, moderate agreement; 0.61–0.80, good or substantial agreement; 0.81–1.00, very good or almost perfect agreement.

**Fig 4:**
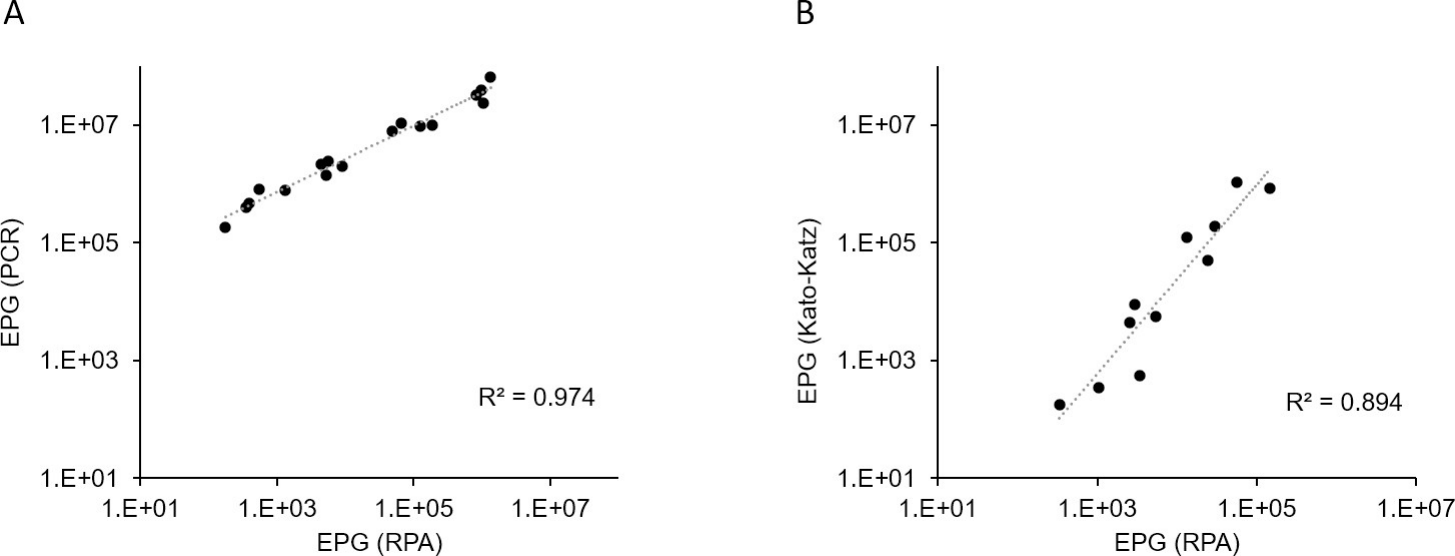
Correlation of the number of *A*. *lumbricoides* eggs per gram (EPG) of stool derived from either Dx4STH-1 and real-time PCR (A) or from Dx4STH-1 and Kato-Katz method (B). Data sets of each assay were generated using the same set of *A*. *lumbricoides*-positive stool samples. For Dx4STH and real-time PCR, EPG was calculated using the standard curve generated from the analysis of the egg-spiked stools. Pearson correlation was used to assess an association between the EPG values derived from the three methods, as well as the RPA T_t_ and PCR C_q_ values and EPG values derived by the Kato–Katz method.

The number of eggs of *A*. *lumbricoides* in infected stools estimated using Dx4STH-1 showed good correlation when compared against EPG based on PCR assay (Pearson’s *r* = 0.9416) (Fig 4A) and KK method (Pearson’s *r* = 0.8104) (Fig 4B). Because of the unavailability of eggs from *T*. *trichiura* and the hookworm species to establish a standard curve, and the limited information on the number of RPA target copies present in their eggs, we were unable to estimate the number of eggs present in stool. Instead, we compared the outputs from RPA with real-time PCR results using the same set of stool samples with different infection intensities. The RPA T_t_ and PCR C_q_ values exhibited moderate to good correlation for *T*. *trichiura* (R^2^ = 0.4907, *r =* 0.7005) and hookworm species (R^2^ = 0.4252, *r =* 0.6521), respectively.

**Fig 5:**
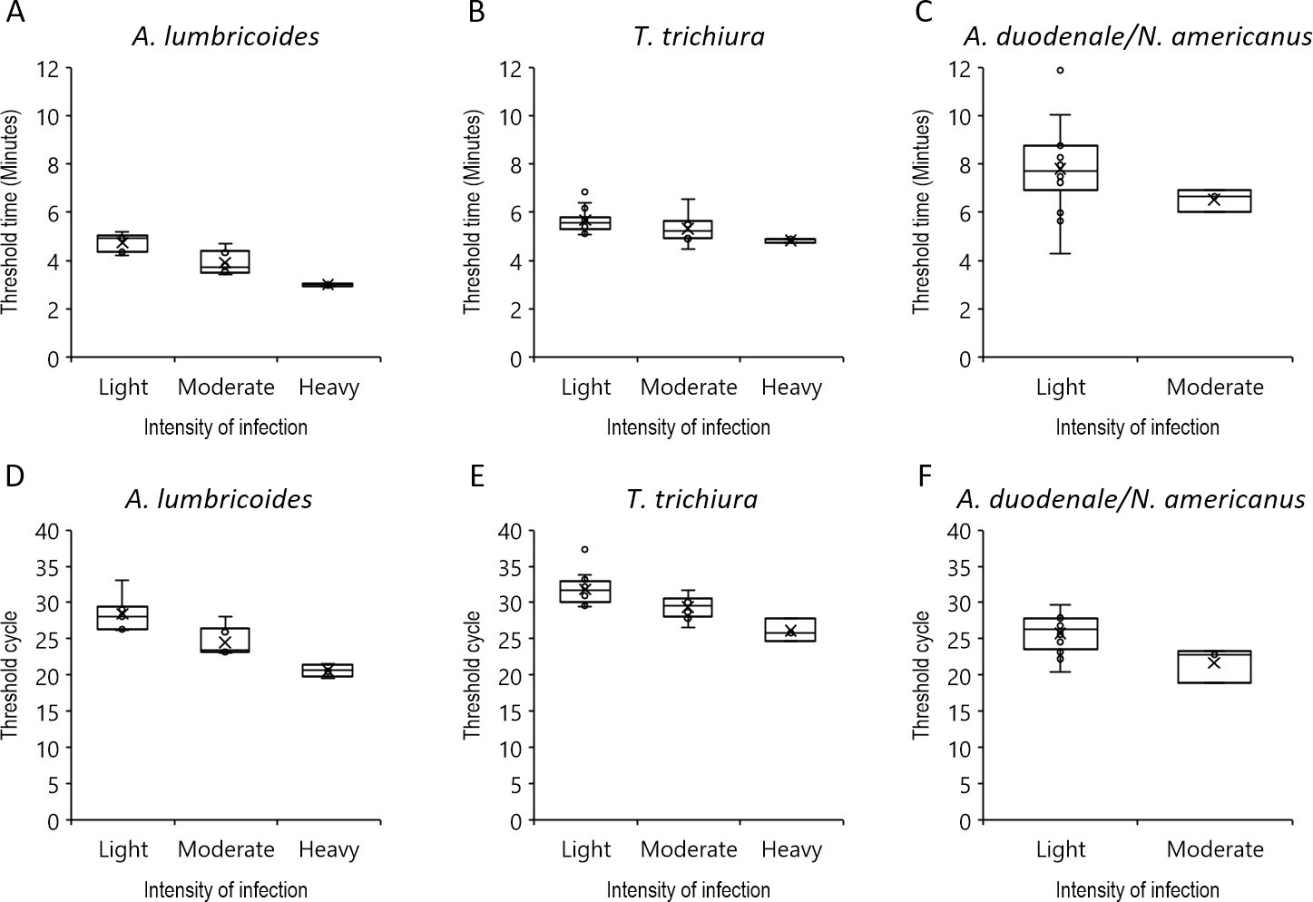
Correlation of RPA T_t_ value or real-time PCR C_q_ and intensity of infection in STH-positive stool samples with known EPG as determined by KK. Data from stool samples that were tested RPA-positive for *A*. *lumbricoides* (n = 21), *T*. *trichiura* (n = 32), and the two hookworms (n = 16). Each DNA extract was analyzed using Dx4STH (A, B and C) and real-time PCR assays (D, E and F). Threshold values were plotted against infection intensities as per KK method in a box and whisker diagram to indicate the median threshold value and variability.

When the RPA T_t_ values were plotted based on infection intensities as determined by KK, the median T_t_ can be distinguished from high-, medium-, and low-intensity infections (Fig 5A, 5B, and 5C). For instance, samples infected with *A*. *lumbricoides* at light intensity exhibited a median T_t_ of 5 minutes, whereas moderate and heavy infections had a median T_t_ of 4 and 3 minutes, respectively. Although T_t_ values for *T*. *trichiura*-positive samples were not well-resolved for light and moderate infection intensities, they were distinguishable from heavy intensities. Median threshold values for light and moderate intensities of hookworm infections were higher than those for corresponding intensities in the other two species. Similar results were obtained when PCR C_q_ values were plotted with infection intensities (Fig 5D, 5E, and 5F).

## Discussion

Diagnostic tools are critical for mapping STH infections, designing control programs, and monitoring program impact [5,47]. Diagnostic tools that are more sensitive than current microscopy methods are needed, particularly after successful MDA when the prevalence and intensity of infection are generally low. These new tools will help control programs determine whether goals have been achieved and whether and when to reduce or discontinue MDA.

We developed a new molecular diagnostic tool for each major STH species by using RPA and then evaluated the tools using a panel of stool samples from STH-endemic regions. We chose RPA over other isothermal NAATs because of its simplicity (few and simple hands-on manipulation steps), speed (results in 5 to 20 min), flexibility (commercial kits available for various detection formats) [48], and ease of implementation in low-resource settings (low temperature requirement necessitates only a simple heating device to achieve accurate detection) [28,29,49]. For each species of STH, RPA primers and fluorescent detection probes target the same DNA regions used in real-time PCR assays [15–17,41]. Each set of optimal primers and probe, identified through extensive screening using very low levels of plasmid DNA standard, specifically detected the target sequence in individual and mixed STH DNAs without non-specific amplification with the DNA from other NTEPs. All four RPA assays were rapid, with fluorescent amplification signals observed within 10 min of incubation at 40°C and detected very low levels of target (<20 target copies per reaction) even in the presence of a high level of background stool DNA.

Simultaneous detection and differentiation of all four STH species is always desirable when multiple samples are being analyzed during STH surveillance [10] because this will reduce analysis time and assay cost. RPA assays for multiple targets using different detection mechanisms have been reported previously [26,50–54]. Real-time fluorescence detection-based RPA assays are superior to end-point detection by lateral flow strips, as the latter method requires additional testing costs and time (i.e., material costs for lateral flow strips, processing steps to dilute and transfer amplicons into lateral flow device/strips, and extra time to visualize results) [55,56]. However, development of a highly multiplexed fluorescent-based RPA assay for detecting all four STH species in a single reaction is limited by the availability of isothermal amplification devices with multiple fluorescent detectors capable of detecting more than three targets (i.e., ESEQuant TS2, Qiagen). To ensure that the new STH diagnostics are not dependent on a device with more than two fluorescent channels, we integrated RPA reactions into duplex/ triplex assays and demonstrated simultaneous and differential detection of *A*. *lumbricoides* and *T*. *trichiura* (Dx4STH-1) and *A*. *duodenale* and *N*. *americanus* (Dx4STH-2).

The performance of Dx4STH-1 and Dx4STH-2 in detecting the four STH species is equivalent to corresponding assays in singleplex format in terms of the limit of detection and specific detection of target when assessed using plasmid DNA standards. The RPA assays in either duplex/ triplex or singleplex formats detected < 50 target copies in the presence of background stool DNA. When the efficacy of the duplex/ triplex RPA assays was compared against results with real-time PCR as comparator method for testing stool samples, Dx4STH-1 was as sensitive and specific as real-time PCR in detecting *A*. *lumbricoides*. Some false-negative and false-positive results were noted with the RPA assays. For hookworms, the two false-positive samples were also positive by KK and were characterized as having light intensity of infection. We suspect that these two samples contained a high level of confounders that inhibited PCR but not RPA, as evidenced by the higher C_q_ of the IC. But since PCR was used as the gold standard, these samples were scored as RPA negatives. One *T*. *trichiura* sample that was missed by RPA had a light intensity of infection based on KK data and a weak PCR signal (C_q_ 37). We suspect that *T*. *trichiura* DNA was in such a small amount because of incomplete lysis of the already small number of *T*. *trichiura* eggs [57] that it was missed by RPA but not PCR. When a limited number of DNA target molecules exist in the sample, the primers may not consistently find and hybridize to their DNA targets present in the amplification reaction (stochastic or random variation) [58]. Along with detecting individual target organisms, the multiplex RPA approach detected three samples with multiple infections, and the results were confirmed by real-time PCR. These samples were found to be infected by either *Ascaris*, *Trichuris*, or *Ascaris* and hookworm. Thus, the use of RPA and PCR to detect coinfection with other STH species revealed an increased prevalence of polyparasitism.

Another important factor to consider for a new STH diagnostic is the ability to quantify the number of targets in the sample, which makes real-time PCR-based assays good candidates. There are a lack of studies describing quantitative RPA, although recent studies have described a strong correlation between real-time RPA and real-time PCR results for various types of animal viruses [30,37,59–64]. From an assay design standpoint, we have shown that each RPA assay produced consistent and linear amplification across a range of target concentrations. In some cases, we noted relatively high CVs in replicate reactions with very low template amount, which is not surprising. This observation may be due to the mixing step at 4 minutes after the reaction has started, which was difficult to maintain between runs or between users. Our data on *A*. *lumbricoides* also showed that the EPG obtained by RPA using T_t_ exhibited a highly significant correlation with the EPG as measured by real-time PCR and the KK method. For *T*. *trichiura* and the hookworms, by contrast, the T_t_ values reflecting parasite-specific DNA loads had a moderate correlation with the egg counts estimated by the KK method. This observation is not surprising and is consistent with previous findings wherein a broad range of PCR C_q_ values have been reported for each KK-determined EPG value [17].

Furthermore, the median T_t_ of positive samples was distinguishable and correlated well in high, medium, and light intensity of infections. In some instances, the T_t_ values for *T*. *trichiura*-positive samples were not well-resolved for light and moderate infection intensities but were distinguishable from heavy intensities. This is probably because RPA reactions occur rapidly and the T_t_ values of samples with varying concentrations may not be resolved well. Further assay optimization may be required to improve separation of T_t_ by staggering the reaction (through lowering incubation temperature, optimizing concentration of RPA reagents and/or Mg acetate) to clearly differentiate the three infection intensities.

Overall, RPA demonstrated performance comparable to that of real-time PCR in detecting *A*. *lumbricoides*, *T*. *trichiura*, and two species of hookworms in stools and provided better results than KK. The fact that the results from RPA and real-time PCR are highly correlated indicates that target detection by RPA assays is possible in a manner that is analogous to real-time PCR. Pending availability of proper tools and instrumentations, RPA assays may provide robust, reliable detection with potential for semi-quantification of STH DNA targets as an alternative indicator to counts of helminth eggs for assessing infection intensity.

### Advantages of RPA in STH surveillance

The RPA assay provides a sensitive diagnostic tool for detecting STH DNA and is a promising technology for use in low-resource settings. The tool enables simultaneous detection of multiple targets with unknown concentrations, similar to traditional real-time PCR. The reagents are available in lyophilized form and are stable for use in field settings. Because the reaction is isothermal, the RPA assay requires only simple or portable instrumentation, and amplification occurs within 5 to 15 minutes, enabling rapid results. Furthermore, the RPA assay design is simpler than other isothermal NAATs because the oligonucleotide design requires only three conserved regions. Its multiplexing capability permitted the addition of an internal control [42] for assessing potential inhibitors in the samples and thereby minimized false negative results without affecting each assay’s performance. As a molecular tool, RPA can detect helminth DNA in stool even without the visible presence of eggs. STH DNA from continuous worm shedding or from eggs that have been lysed is co-extracted, resulting in amplification of their DNA, and could increase parasite detection. Assuming optimal workflow and availability of at least four 8-tube isothermal devices, roughly 100 samples can be processed and analyzed in 4.8 to 6 hours depending on which sample extraction methodology is used [8].

STH prevalence is inaccurately estimated by current coproscopic tools, particularly in regions with low prevalence. More sensitive molecular tools such as RPA can more accurately assess infection prevalence and intensity following MDA and thereby improve MDA decisions and advance STH control.

### Remaining barriers to scale-up of RPA products

Despite proving effective for parasite detection and quantification of infection intensity, multiplex RPA assays have several limitations. The cost is still high at around US $9–10 per test including the sample extraction step [8], which may prevent its uptake by control programs in low-resource settings. Substantially reducing the cost would enable broader use in affected countries. Molecular tools such as RPA or PCR may be unable to differentiate residual DNA from previous infections (i.e., worm shedding or dead parasites) from DNA from current infections (DNA from eggs), which may pose a challenge in interpreting prevalence and intensity estimates. Stool is currently the only valid sample because of the biology of the STH infection. Effective stool DNA extraction methods currently available are not suitable for field use (e.g., they require use of a centrifuge). A simple alkaline lysis treatment is an inexpensive and efficient method to release DNA and is compatible with RPA without further clean-up due to the high tolerance of RPA reagents to inhibitors. However, this treatment also dilutes the STH targets that may already be present in low amounts. To obtain detection, a DNA concentration method without additional centrifugation steps will maximize its use in low-resource settings. A magnetic bead-based strategy may be useful for nucleic acid extraction in the field, though this approach requires further evaluation and optimization (S2 Table). Another barrier is the availability of isothermal devices with multiple detectors for multiplexing, and automated mixing capability to allow a more consistent results thus providing quantitative data similar to qPCR machines. Also, the correlation of DNA levels with the numbers of eggs in *T*. *trichiura* and hookworm infections was not determined due to the unavailability of purified eggs of these species. DNA output from any molecular tests has not been translated yet to intensity of infection, and further work is needed to compare egg counts with the DNA concentration or target copies per egg. A potential issue related to this comparison is the increase in copies of DNA once eggs have embryonated, although previous work on *Ascaris* showed that ITS-1 rDNA levels were proportional to egg cell numbers, which increase as eggs developed from single cells to mature larvae and ultimately reaching a constant level per egg [65], suggesting that accurate quantification is feasible. Lastly, the RPA assays detected or could potentially detect closely-related species due to the conservative nature of the target region. The Al-RPA was demonstrated to detect *A*. *suum* DNA, while BLAST revealed that Ad-RPA primiers and probes could also detect *A*. *ceylanicum* and *A*. *caninum*.

## Supporting information

S1 Table Primers and probes used for real time PCR analysis of soil-transmitted helminths. (DOCX)

S2 Table Comparison of modified alkaline lysis–magnetic bead (MAL-MB) based stool DNA extraction versus three commercial stool DNA extraction kits. (DOCX)

## References

[1] Yap P, Furst T, Muller I, Kriemler S, Utzinger J, Steinmann P. Determining soil-transmitted helminth infection status and physical fitness of school-aged children. Journal of Visualized Experiments: JoVE. 2012(66):e3966. doi: 10.3791/3966 22951972 PMC3486755

[2] Hu Y, Ellis BL, Yiu YY, Miller MM, Urban JF, Shi LZ, et al. An extensive comparison of the effect of anthelmintic classes on diverse nematodes. PLoS One. 2013;8(7):e70702. doi: 10.1371/journal.pone.0070702 23869246 PMC3712009

[3] Scholte RG, Schur N, Bavia ME, Carvalho EM, Chammartin F, Utzinger J, et al. Spatial analysis and risk mapping of soil-transmitted helminth infections in Brazil, using Bayesian geostatistical models. GeospatHealth. 2013;8(1):97–110. doi: 10.4081/gh.2013.58 24258887

[4] WHO. Eliminating soil-transmitted helminthiasis as a public health problem in children: Progress report 2001–2010 and strategic plan 2011–2020. World Health Organization, Geneva, Switzerland; 2012 2012.

[5] Bergquist R, Johansen MV, Utzinger J. Diagnostic dilemmas in helminthology: what tools to use and when? Trends Parasitol. 2009;25(4):151–6. doi: 10.1016/j.pt.2009.01.004 19269899

[6] WHO. Assessing the epidemiology of soil-transmitted helminths during a transmission assessment survey (TAS) in the global programme for the elimination of lymphatic filariasis. Geneva, Switzerland: World Health Organization, diseases Docont; 2015. Report No.: ISBN: 978 92 4 150838 4 Contract No.: WHO/HTM/NTD/PCT/2015.2.

[7] Harhay MO, Horton J, Olliaro PL, Utzinger J. Diagnostics are central for a truly holistic approach against intestinal parasitic diseases. Int J Infect Dis. 2011;15(2):e76–7. doi: 10.1016/j.ijid.2010.10.007 21145770

[8] Storey HL, Agarwal N, Cantera J, Golden A, Gallo K, Herrick T, et al. Formative research to inform development of a new diagnostic for soil-transmitted helminths: Going beyond the laboratory to ensure access to a needed product. PLoS Negl Trop Dis. 2019;13(5):e0007372. doi: 10.1371/journal.pntd.0007372 31150389 PMC6561600

[9] Hawkins KR, Cantera JL, Storey HL, Leader BT, de Los Santos T. Diagnostic tests to support late-stage control programs for schistosomiasis and soil-transmitted helminthiases. PLoS Negl Trop Dis. 2016;10(12):e0004985. doi: 10.1371/journal.pntd.0004985 28005900 PMC5179049

[10] WHO. Assessing the epidemiology of soil-transmitted helminths during a transmission assessment survey in the Global programme to Eliminate Lymphatic Filariasis. Geneva, Switzerland; 2015 2015.

[11] arcon de NB, Ruiz R, Losada S, Colmenares C, Contreras R, Cesari IM, et al. Detection of schistosomiasis cases in low-transmission areas based on coprologic and serologic criteria The Venezuelan experience. Acta Trop. 2007;103(1):41–9. doi: 10.1016/j.actatropica.2007.04.018 17606217

[12] Booth M, Vounatsou P, N’Goran EK, Tanner M, Utzinger J. The influence of sampling effort and the performance of the Kato-Katz technique in diagnosing Schistosoma mansoni and hookworm co-infections in rural Cote d’Ivoire. Parasitology. 2003;127(Pt 6):525–31. doi: 10.1017/s0031182003004128 14700188

[13] Burlandy-Soares LC, de Souza Dias LC, Kanamura HY, de Oliveira EJ, Ciaravolo RM. Schistosomiasis mansoni: follow-up of control program based on parasitologic and serologic methods in a Brazilian community of low endemicity. Mem Inst Oswaldo Cruz. 2003;98(6):853–9. doi: 10.1590/s0074-02762003000600025 14595468

[14] Guyatt HL, Bundy DA. Estimating prevalence of community morbidity due to intestinal helminths: prevalence of infection as an indicator of the prevalence of disease. Trans R Soc Trop Med Hyg. 1991;85(6):778–82. doi: 10.1016/0035-9203(91)90453-6 1801353

[15] Mejia R, Vicuna Y, Broncano N, Sandoval C, Vaca M, Chico M, et al. A novel, multi-parallel, real-time polymerase chain reaction approach for eight gastrointestinal parasites provides improved diagnostic capabilities to resource-limited at-risk populations. Am J Trop Med Hyg. 2013;88(6):1041–7. doi: 10.4269/ajtmh.12-0726 23509117 PMC3752800

[16] Basuni M, Muhi J, Othman N, Verweij JJ, Ahmad M, Miswan N, et al. A pentaplex real-time polymerase chain reaction assay for detection of four species of soil-transmitted helminths. Am J Trop Med Hyg. 2011;84(2):338–43. doi: 10.4269/ajtmh.2011.10-0499 21292911 PMC3029194

[17] Verweij JJ, Brienen EA, Ziem J, Yelifari L, Polderman AM, van LL. Simultaneous detection and quantification of Ancylostoma duodenale, Necator americanus, and Oesophagostomum bifurcum in fecal samples using multiplex real-time PCR. Am J Trop Med Hyg. 2007;77(4):685–90. 17978072

[18] Pilotte N, Papaiakovou M, Grant JR, Bierwert LA, Llewellyn S, McCarthy JS, et al. Improved PCR-based detection of soil transmitted helminth infections using a next-generation sequencing approach to assay design. PLoS Negl Trop Dis. 2016;10(3):e0004578. doi: 10.1371/journal.pntd.0004578 27027771 PMC4814118

[19] Llewellyn S, Inpankaew T, Nery SV, Gray DJ, Verweij JJ, Clements AC, et al. Application of a multiplex quantitative PCR to assess prevalence and intensity of intestinal parasite infections in a controlled clinical trial. PLoS Negl Trop Dis. 2016;10(1):e0004380. doi: 10.1371/journal.pntd.0004380 26820626 PMC4731196

[20] Basuni M, Mohamed Z, Ahmad M, Zakaria NZ, Noordin R. Detection of selected intestinal helminths and protozoa at Hospital Universiti Sains Malaysia using multiplex real-time PCR. Trop Biomed. 2012;29(3):434–42. 23018507

[21] Gordon CA, McManus DP, Acosta LP, Olveda RM, Williams GM, Ross AG, et al. Multiplex real-time PCR monitoring of intestinal helminths in humans reveals widespread polyparasitism in Northern Samar, the Philippines. Int J Parasitol. 2015;45(7):477–83. doi: 10.1016/j.ijpara.2015.02.011 25858090

[22] Shiraho EA, Eric AL, Mwangi IN, Maina GM, Kinuthia JM, Mutuku MW, et al. Development of a Loop Mediated Isothermal Amplification for Diagnosis of Ascaris lumbricoides in Fecal Samples. J Parasitol Res. 2016;2016:7376207. doi: 10.1155/2016/7376207 27882242 PMC5108867

[23] Mugambi RM, Agola EL, Mwangi IN, Kinyua J, Shiraho EA, Mkoji GM. Development and evaluation of a Loop Mediated Isothermal Amplification (LAMP) technique for the detection of hookworm (Necator americanus) infection in fecal samples. Parasites & Vectors. 2015;8(1):574. doi: 10.1186/s13071-015-1183-9 26546069 PMC4636844

[24] Watts MR, James G, Sultana Y, Ginn AN, Outhred AC, Kong F, et al. A loop-mediated isothermal amplification (LAMP) assay for Strongyloides stercoralis in stool that uses a visual detection method with SYTO-82 fluorescent dye. Am J Trop Med Hyg. 2014;90(2):306–11. doi: 10.4269/ajtmh.13-0583 24323513 PMC3919238

[25] Rashwan N, Diawara A, Scott ME, Prichard RK. Isothermal diagnostic assays for the detection of soil-transmitted helminths based on the SmartAmp2 method. Parasit Vectors. 2017;10(1):496. doi: 10.1186/s13071-017-2420-1 29047387 PMC5648480

[26] Piepenburg O, Williams CH, Stemple DL, Armes NA. DNA detection using recombination proteins. PLoS Biol. 2006;4(7):e204. doi: 10.1371/journal.pbio.0040204 16756388 PMC1475771

[27] Lillis L, Siverson J, Lee A, Cantera J, Parker M, Piepenburg O, et al. Factors influencing recombinase polymerase amplification (RPA) assay outcomes at point of care. Mol Cell Probes. 2016;30(2):74–8. doi: 10.1016/j.mcp.2016.01.009 26854117 PMC4818709

[28] Lillis L, Lehman D, Singhal MC, Cantera J, Singleton J, Labarre P, et al. Non-instrumented incubation of a recombinase polymerase amplification assay for the rapid and sensitive detection of proviral HIV-1 DNA. PLoS One. 2014;9(9):e108189. doi: 10.1371/journal.pone.0108189 25264766 PMC4180440

[29] Wang R, Zhang F, Wang L, Qian W, Qian C, Wu J, et al. Instant, visual, and instrument-free method for on-site screening of GTS 40-3-2 soybean based on body-heat triggered recombinase polymerase amplification. Anal Chem. 2017;89(8):4413–8. doi: 10.1021/acs.analchem.7b00964 28345860

[30] Moore MD, Jaykus LA. Development of a recombinase polymerase amplification assay for detection of epidemic human noroviruses. Sci Rep. 2017;7:40244. doi: 10.1038/srep40244 28067278 PMC5220337

[31] Crannell ZA, Rohrman B, Richards-Kortum R. Development of a quantitative recombinase polymerase amplification assay with an internal positive control. J Vis Exp. 2015(97). doi: 10.3791/52620 25867513 PMC4401391

[32] Crannell ZA, Rohrman B, Richards-Kortum R. Quantification of HIV-1 DNA using real-time recombinase polymerase amplification. Anal Chem. 2014;86(12):5615–9. doi: 10.1021/ac5011298 24873435

[33] Crannell ZA, Cabada MM, Castellanos-Gonzalez A, Irani A, White AC, Richards-Kortum R. Recombinase polymerase amplification-based assay to diagnose Giardia in stool samples. Am J Trop Med Hyg. 2014. doi: 10.4269/ajtmh.14-0593 25510713 PMC4350554

[34] Wu YD, Xu MJ, Wang QQ, Zhou CX, Wang M, Zhu XQ, et al. Recombinase polymerase amplification (RPA) combined with lateral flow (LF) strip for detection of Toxoplasma gondii in the environment. Vet Parasitol. 2017;243:199–203. doi: 10.1016/j.vetpar.2017.06.026 28807294

[35] Xing W, Yu X, Feng J, Sun K, Fu W, Wang Y, et al. Field evaluation of a recombinase polymerase amplification assay for the diagnosis of Schistosoma japonicum infection in Hunan province of China. BMC Infect Dis. 2017;17(1):164. doi: 10.1186/s12879-017-2182-6 28222680 PMC5320755

[36] Rostron P, Pennance T, Bakar F, Rollinson D, Knopp S, Allan F, et al. Development of a recombinase polymerase amplification (RPA) fluorescence assay for the detection of Schistosoma haematobium. Parasit Vectors. 2019;12(1):514. doi: 10.1186/s13071-019-3755-6 31685024 PMC6827214

[37] Cabada MM, Malaga JL, Castellanos-Gonzalez A, Bagwell KA, Naeger PA, Rogers HK, et al. Recombinase polymerase amplification compared to real-time polymerase chain reaction test for the detection of Fasciola hepatica in human stool. Am J Trop Med Hyg. 2017;96(2):341–6. doi: 10.4269/ajtmh.16-0601 27821691 PMC5303034

[38] Patel P, Abd El Wahed A, Faye O, Pruger P, Kaiser M, Thaloengsok S, et al. A field-deployable reverse transcription recombinase polymerase amplification assay for rapid detection of the chikungunya virus. PLoS Negl Trop Dis. 2016;10(9):e0004953. doi: 10.1371/journal.pntd.0004953 27685649 PMC5042537

[39] Saldarriaga OA, Castellanos-Gonzalez A, Porrozzi R, Baldeviano GC, Lescano AG, de Los Santos MB, et al. An innovative field-applicable molecular test to diagnose cutaneous Leishmania viannia spp. infections. PLoS Negl Trop Dis. 2016;10(4):e0004638. doi: 10.1371/journal.pntd.0004638 27115155 PMC4845993

[40] Mondal D, Ghosh P, Khan MA, Hossain F, Bohlken-Fascher S, Matlashewski G, et al. Mobile suitcase laboratory for rapid detection of Leishmania donovani using recombinase polymerase amplification assay. Parasit Vectors. 2016;9(1):281. doi: 10.1186/s13071-016-1572-8 27177926 PMC4868004

[41] Easton AV, Oliveira RG, O’Connell EM, Kepha S, Mwandawiro CS, Njenga SM, et al. Multi-parallel qPCR provides increased sensitivity and diagnostic breadth for gastrointestinal parasites of humans: field-based inferences on the impact of mass deworming. Parasit Vectors. 2016;9:38. doi: 10.1186/s13071-016-1314-y 26813411 PMC4729172

[42] Hill-Cawthorne GA, Hudson LO, El Ghany MFA, Piepenburg O, Nair M, Dodgson A, et al. Recombinations in Staphylococcal Cassette Chromosome mec Elements Compromise the Molecular Detection of Methicillin Resistance in Staphylococcus aureus. PLOS ONE. 2014;9(6):e101419. doi: 10.1371/journal.pone.0101419 24972080 PMC4074205

[43] Leles D, Gardner SL, Reinhard K, Iñiguez A, Araujo A. Are Ascaris lumbricoides and Ascaris suum a single species? Parasites & Vectors. 2012;5(1):42. doi: 10.1186/1756-3305-5-42 22348306 PMC3293767

[44] U.S. Department of Health and Human Services FaDA. Statistical guidance on reporting results from studies evaluating diagnostic tests. Rockville, MD: U.S. Department of Health and Human Services; 2007. p. 39p.

[45] Chen J, Kadlubar FF, Chen JZ. DNA supercoiling suppresses real-time PCR: a new approach to the quantification of mitochondrial DNA damage and repair. Nucleic acids research. 2007;35(4):1377–88. doi: 10.1093/nar/gkm010 17284464 PMC1851651

[46] Hou Y, Zhang H, Miranda L, Lin S. Serious overestimation in quantitative PCR by circular (supercoiled) plasmid standard: microalgal pcna as the model gene. PloS one. 2010;5(3):e9545–e. doi: 10.1371/journal.pone.0009545 20221433 PMC2832698

[47] McCarthy JS, Lustigman S, Yang GJ, Barakat RM, Garcia HH, Sripa B, et al. A research agenda for helminth diseases of humans: diagnostics for control and elimination programmes. PLoSNeglTropDis. 2012;6(4):e1601. doi: 10.1371/journal.pntd.0001601 22545166 PMC3335877

[48] Daher RK, Stewart G, Boissinot M, Bergeron MG. Recombinase polymerase amplification for diagnostic applications. Clin Chem. 2016;62(7):947–58. doi: 10.1373/clinchem.2015.245829 27160000 PMC7108464

[49] Crannell ZA, Rohrman B, Richards-Kortum R. Equipment-free incubation of recombinase polymerase amplification reactions using body heat. PLoS One. 2014;9(11):e112146. doi: 10.1371/journal.pone.0112146 25372030 PMC4221156

[50] Lau HY, Wang Y, Wee EJ, Botella JR, Trau M. Field demonstration of a multiplexed point-of-care diagnostic platform for plant pathogens. Anal Chem. 2016;88(16):8074–81. doi: 10.1021/acs.analchem.6b01551 27403651

[51] Kersting S, Rausch V, Bier FF, von Nickisch-Rosenegk M. Multiplex isothermal solid-phase recombinase polymerase amplification for the specific and fast DNA-based detection of three bacterial pathogens. Mikrochim Acta. 2014;181(13–14):1715–23. doi: 10.1007/s00604-014-1198-5 25253912 PMC4167443

[52] Choi G, Jung JH, Park BH, Oh SJ, Seo JH, Choi JS, et al. A centrifugal direct recombinase polymerase amplification (direct-RPA) microdevice for multiplex and real-time identification of food poisoning bacteria. Lab Chip. 2016;16(12):2309–16. doi: 10.1039/c6lc00329j 27216297

[53] Crannell Z, Castellanos-Gonzalez A, Nair G, Mejia R, White AC, Richards-Kortum R. Multiplexed recombinase polymerase amplification assay to detect intestinal protozoa. Anal Chem. 2016;88(3):1610–6. doi: 10.1021/acs.analchem.5b03267 26669715

[54] Santiago-Felipe S, Tortajada-Genaro LA, Morais S, Puchades R, Maquieira A. Isothermal DNA amplification strategies for duplex microorganism detection. Food Chem. 2015;174C:509–15. doi: 10.1016/j.foodchem.2014.11.080 25529713

[55] Crannell ZA, Cabada MM, Castellanos-Gonzalez A, Irani A, White AC, Richards-Kortum R. Recombinase polymerase amplification-based assay to diagnose Giardia in stool samples. Am J Trop Med Hyg. 2015;92(3):583–7. doi: 10.4269/ajtmh.14-0593 25510713 PMC4350554

[56] Crannell ZA, Castellanos-Gonzalez A, Irani A, Rohrman B, White AC, Richards-Kortum R. Nucleic acid test to diagnose cryptosporidiosis: lab assessment in animal and patient specimens. Anal Chem. 2014;86(5):2565–71. doi: 10.1021/ac403750z 24479858 PMC3958140

[57] Demeler J, Ramunke S, Wolken S, Ianiello D, Rinaldi L, Gahutu JB, et al. Discrimination of gastrointestinal nematode eggs from crude fecal egg preparations by inhibitor-resistant conventional and real-time PCR. PLoS One. 2013;8(4):e61285. doi: 10.1371/journal.pone.0061285 23620739 PMC3631180

[58] Weusten J, Herbergs J. A stochastic model of the processes in PCR based amplification of STR DNA in forensic applications. Forensic Science International: Genetics. 2012;6(1):17–25. doi: 10.1016/j.fsigen.2011.01.003 21295532

[59] Yang Y, Qin X, Zhang X, Zhao Z, Zhang W, Zhu X, et al. Development of real-time and lateral flow dipstick recombinase polymerase amplification assays for rapid detection of goatpox virus and sheeppox virus. Virol J. 2017;14(1):131. doi: 10.1186/s12985-017-0792-7 28716095 PMC5514530

[60] Yang Y, Qin X, Zhang W, Li Z, Zhang S, Li Y, et al. Development of an isothermal recombinase polymerase amplification assay for rapid detection of pseudorabies virus. Mol Cell Probes. 2017;33:32–5. doi: 10.1016/j.mcp.2017.03.005 28342800

[61] Yang Y, Qin X, Sun Y, Cong G, Li Y, Zhang Z. Development of isothermal recombinase polymerase amplification assay for rapid detection of porcine Circovirus Type 2. Biomed Res Int. 2017;2017:8403642. doi: 10.1155/2017/8403642 28424790 PMC5382309

[62] Yang Y, Qin X, Song Y, Zhang W, Hu G, Dou Y, et al. Development of real-time and lateral flow strip reverse transcription recombinase polymerase Amplification assays for rapid detection of peste des petits ruminants virus. Virol J. 2017;14(1):24. doi: 10.1186/s12985-017-0688-6 28173845 PMC5297045

[63] Wang JC, Liu LB, Han QA, Wang JF, Yuan WZ. An exo probe-based recombinase polymerase amplification assay for the rapid detection of porcine parvovirus. J Virol Methods. 2017;248:145–7. doi: 10.1016/j.jviromet.2017.06.011 28690087

[64] Wang J, Liu L, Wang J, Sun X, Yuan W. Recombinase polymerase amplification assay—A simple, fast and cost-effective alternative to real time PCR for specific detection of feline Herpesvirus-1. PLoS One. 2017;12(1):e0166903. doi: 10.1371/journal.pone.0166903 28045956 PMC5207716

[65] Pecson BM, Barrios JA, Johnson DR, Nelson KL. A real-time PCR method for quantifying viable ascaris eggs using the first internally transcribed spacer region of ribosomal DNA. Appl Environ Microbiol. 2006;72(12):7864–72. doi: 10.1128/AEM.01983-06 17056687 PMC1694259

