# Supplementary material for: Sensitive and semiquantitative detection of soil-transmitted helminth infection in stool using a recombinase polymerase amplification-based assay
Source: PLoS Negl Trop Dis. 2021 Sep 13;15(9):e0009782. doi: 10.1371/journal.pntd.0009782 (PMC8459997; doi:10.1371/journal.pntd.0009782)
Supplement: S1 Table — (DOCX) [file pntd.0009782.s001.docx]

**S1 Table.** Primers and probes used for real time PCR analysis of soil-transmitted helminths.

| Target organism | Oligo name | Sequence (5' --> 3') | Nucleotide position | Amplicon size (bp) | Target gene | GenBank acc. No. | Reference |
| --- | --- | --- | --- | --- | --- | --- | --- |
| *Ascaris lumbricoides* | Alum96F | GTAATAGCAGTCGGCGGTTTCTT | 96-118 | 89 | ITS1 | AB571301.1 | [1] |
|  | Alum183R | GCCCAACATGCCACCTATTC | 183-164 |  |  |  |  |
|  | Alum124P | TTGGCGGACAATTGCATGCGAT | 124-145 |  |  |  |  |
| *Trichuris trichiura* | Tt-F_east | TTGCCTGTTGGGTGTATCTGTAA | 71520-71542 | 60 | unknown | HG805809.1 | [2] |
|  | Tt-R_east | TGCTCATCCATCCGTTGGT | 71580-71562 |  |  |  |  |
|  | Tt-P_east | TAAACTTCAAAATGCCC | 71544-71560 |  |  |  |  |
| *Ancylostoma duodenale* | Ad125F | GAATGACAGCAAACTCGTTGTTG | 644-666 | 71 | ITS2 | EU344797.1 | [1,3,4] |
|  | Ad195R | ATACTAGCCACTGCCGAAACGT | 693-714 |  |  |  |  |
|  | Ad155MGB | ATCGTTTACCGACTTTAG | 691-674 |  |  |  |  |
| *Necatur americanus* | Na58F | CTGTTTGTCGAACGGTACTTGC | 58-79 | 101 | ITS2 | AJ001599.1 | [1,3,4] |
|  | Na158R | ATAACAGCGTGCACATGTTGC | 158-138 |  |  |  |  |
|  | Na81MGB | CTGTACTACGCATTGTATAC | 81-100 |  |  |  |  |

The amplification conditions for each assay were verified on a Brilliant II qPCR low ROX master mix (Agilent Technologies, Santa Clara, CA) using the Mx3005P qPCR system (Agilent Technologies). The 25 μL reaction contained 1X master mix, 5.5 mM MgCl_2,_ 150 nM forward and reverse primers each, 200 nM fluorescently labeled hybridization probe, and 2 μL template DNA. Thermocycling conditions consisted of 95°C for 15 min, and 50 cycles of 95°C for 15 sec, 60°C for 30 sec, and 72°C for 30 sec. Fluorescence was measured during the annealing step of each cycle. Each PCR run included a negative control consisting of PCR mix without target DNA. Real-time PCR assay was considered positive if the C_q_ value < 40, and negative if no amplification curve was obtained or when the C_q_ value >40. To ensure there was no inhibition during PCR, cloned *Bacillus subtilis* rpoB plasmid DNA was included as an inhibition control.

**References**

1. Basuni M, Muhi J, Othman N, Verweij JJ, Ahmad M, Miswan N, et al. A pentaplex real-time polymerase chain reaction assay for detection of four species of soil-transmitted helminths. Am J Trop Med Hyg. 2011;84(2):338-43.

2. Easton AV, Oliveira RG, O'Connell EM, Kepha S, Mwandawiro CS, Njenga SM, et al. Multi-parallel qPCR provides increased sensitivity and diagnostic breadth for gastrointestinal parasites of humans: field-based inferences on the impact of mass deworming. Parasit Vectors. 2016;9:38.

3. Verweij JJ, Brienen EA, Ziem J, Yelifari L, Polderman AM, van LL. Simultaneous detection and quantification of Ancylostoma duodenale, Necator americanus, and Oesophagostomum bifurcum in fecal samples using multiplex real-time PCR. Am J Trop Med Hyg. 2007;77(4):685-90.

4. Mejia R, Vicuna Y, Broncano N, Sandoval C, Vaca M, Chico M, et al. A novel, multi-parallel, real-time polymerase chain reaction approach for eight gastrointestinal parasites provides improved diagnostic capabilities to resource-limited at-risk populations. Am J Trop Med Hyg. 2013;88(6):1041-7.
