# Supplementary material for: Sensitive and semiquantitative detection of soil-transmitted helminth infection in stool using a recombinase polymerase amplification-based assay
Source: PLoS Negl Trop Dis. 2021 Sep 13;15(9):e0009782. doi: 10.1371/journal.pntd.0009782 (PMC8459997; doi:10.1371/journal.pntd.0009782)
Supplement: S2 Table — (DOCX) [file pntd.0009782.s002.docx]

**S2 Table.** Comparison of modified alkaline lysis – magnetic bead (MAL-MB) based stool DNA extraction versus three commercial stool DNA extraction kits. 200-mg stool aliquots (BioIVT) were spiked with *Ascaris suum* eggs (Excelsior Sentinel, Inc) at different levels (EPG) corresponding to light and moderate infection intensities. The DNA was extracted using MAL–MB and benchmarked against PowerFecal (MO BIO) and QIAamp (QIAGEN) stool DNA extraction kits. The extracts were tested using Al-RPA. MAL–MB showed no significant difference with MOBIO (T-test, P=0.062), while MAL–MB performed better than QIAGEN (T-test, P =<0.001) in terms of the number of Al-RPA positives produced.

| ***A. suum* eggs spiked in stool (EPG)** | **Number of Al-RPA positives/ total number of spike stools** | | |
| --- | --- | --- | --- |
|  | **MAL-MB** | **PowerFecal** | **QIAamp** |
| 15000 | 10/10 | *n.d.* | 10/10 |
| 5000 | 10/10 | 6/6 | 15/16 |
| 250 | 10/10 | 6/6 | 6/20 |
| 125 | 8/10 | 6/6 | 2/20 |
| 50 | 10/10 | 3/6 | 4/20 |
| 25 | 9/10 | 3/6 | *n.d.* |
| 0 | 0/6 | 0/6 | 0/10 |
